# Retraction: MiR-365a-3p-mediated regulation of HELLS/GLUT1 axis suppresses aerobic glycolysis and gastric cancer growth

**DOI:** 10.3389/fonc.2024.1519833

**Published:** 2024-11-12

**Authors:** Frontiers Editorial Office

Following publication, concerns were raised regarding the integrity of the images in the published figures. Image duplication concerns were identified in Figure 2E, 3E, and 5G.

Frontiers conducted an investigation in accordance with Frontiers’ policies. As a result, the data and conclusions of the article have been deemed unreliable and the article has been retracted. The authors notified Frontiers of the image concerns and have agreed to a retraction.

